# Temporal Muscle Thickness as a Prognostic Marker in a Real‐Life Cohort of Newly Diagnosed *MGMT* Promoter Methylated Glioblastoma: A Multicentric Imaging Analysis

**DOI:** 10.1002/cam4.70689

**Published:** 2025-04-22

**Authors:** Lazaros Lazaridis, Christoph Moenninghoff, Elisabeth Bumes, Dorothee Cäcilia Spille, Michael Müther, Tim Schulz, Sina Heider, Sarina Agkatsev, Teresa Schmidt, Tobias Blau, Christoph Oster, Walter Stummer, Almuth Friederike Kessler, Clemens Seidel, Oliver Grauer, Peter Hau, Yahya Ahmadipour, Ulrich Sure, Kathy Keyvani, Ulrich Herrlinger, Christoph Kleinschnitz, Martin Stuschke, Nika Guberina, Ken Herrmann, Cornelius Deuschl, Björn Scheffler, Sied Kebir, Martin Glas

**Affiliations:** ^1^ Department of Neurology and Center for Translational Neuro‐ and Behavioral Sciences (C‐TNBS), Division of Clinical Neurooncology, University Medicine Essen University Duisburg‐Essen Essen Germany; ^2^ German Cancer Consortium (DKTK) Partner Site University Medicine Essen Essen Germany; ^3^ Department of Neurology, University Hospital Knappschaftskrankenhaus Bochum Ruhr University Bochum Bochum Germany; ^4^ University Institute for Radiology, Neuroradiology and Nuclear Medicine Johannes Wesling Klinikum Minden Minden Germany; ^5^ Department of Neurology and Wilhelm Sander‐NeuroOncology Unit University Hospital Regensburg Regensburg Germany; ^6^ Department of Neurosurgery University Hospital Münster Münster Germany; ^7^ Department of Neurosurgery University Hospital of Würzburg Würzburg Germany; ^8^ Department of Radiotherapy and Radiation Oncology University Hospital Leipzig Leipzig Germany; ^9^ Institute of Neuropathology, University Medicine Essen University Duisburg‐Essen Essen Germany; ^10^ DKFZ Division Translational Neurooncology at the WTZ and German Cancer Consortium (DKTK) Partner site Essen/Düsseldorf, a partnership between DKFZ and University Hospital Essen Germany; ^11^ Department of Neurology with Institute of Translational Neurology Münster Germany; ^12^ Department of Neurosurgery and Spine Surgery University Medicine Essen, University Duisburg‐Essen Essen Germany; ^13^ Division of Clinical Neurooncology, Department of Neurology and Center for Integrated Oncology University Hospital Bonn Bonn Germany; ^14^ Department of Radiotherapy University Medicine Essen Essen Germany; ^15^ Department of Nuclear Medicine, University Medicine Essen University Duisburg‐Essen Essen Germany; ^16^ Institute for Diagnostic and Interventional Radiology and Neuroradiology, University Medicine Essen University Duisburg‐Essen Essen Germany; ^17^ Department of Neurology and Neurooncology St. Marien Hospital Lünen Lünen Germany

**Keywords:** CeTeG, glioblastoma, imaging, *MGMT*, temporal muscle thickness

## Abstract

**Introduction:**

Prior research has identified temporal muscle thickness (TMT) as a prognostic marker in glioblastoma. Nonetheless, implementation in daily clinical practice is complicated due to the heterogeneity of previous studies. We performed a multicentric analysis aiming to validate recently proposed sex‐specific cutoff values using a homogeneous cohort of newly diagnosed *MGMT* promoter methylated glioblastoma patients; we included a balanced control cohort for comparison.

**Materials and Methods:**

TMT was measured at baseline using the initial preoperative/postoperative magnetic resonance images (MRIs) and in disease course using the first MRI after radiotherapy. Patients were divided by sex and TMT into “at risk of sarcopenia” or “normal muscle status.” Kaplan–Meier and multivariable Cox regression analysis was used for survival correlation.

**Results:**

In total, *n* = 126 patients were included (*n* = 66 treated with CCNU/temozolomide, *n* = 60 with single‐drug temozolomide). Patients with normal muscle mass at baseline had significantly prolonged survival (median overall survival: 44.2 months versus 16.7 months with CCNU/temozolomide, and 29.5 months versus 17.4 months with single‐drug temozolomide) compared to those at risk of sarcopenia. In a multivariable Cox regression analysis, normal muscle mass and an initial age at diagnosis of < 50 years emerged as significant prognostic markers. Longitudinally, survival was longest in patients with lack of TMT decline over the disease course.

**Discussion:**

This analysis confirms TMT as an important prognostic marker in glioblastoma in two real‐life cohorts. However, in order to establish TMT assessment as a routine marker for patient selection and therapeutic measures, further validation in prospective controlled trials is necessary.

## 1 Introduction

Prognosis of glioblastoma remains unfavorable despite recent progress in understanding the biology of primary brain tumors [1]. Almost every patient experiences disease relapse after standard‐of‐care therapy, which includes surgical resection, radiotherapy as well as chemotherapy [2]. Strict patient selection is crucial to avoid ineffectiveness of treatment as well as unnecessary adverse events. In the CeTeG/NOA‐09 randomized phase 3 trial, for instance, combined treatment with temozolomide and CCNU resulted in a significantly prolonged median overall survival (mOS) compared to temozolomide treatment alone in newly diagnosed glioblastoma patients ≤ 70 years of age harboring the prognostically favorable methylation of the O(6)‐methylguanine‐DNA methyltransferase (*MGMT*) promoter [3]. Additionally, in a multicentric real‐life analysis, a Karnofsky performance score (KPS) ≥ 90% emerged to be an independent prognostic factor for progression‐free and overall survival (PFS and OS) for the treatment with combined temozolomide and CCNU in newly diagnosed *MGMT* promoter methylated glioblastoma [4].

In general, patients' frailty is a known key factor negatively affecting survival [5]. Temporal muscle thickness (TMT) assessed in cranial magnetic resonance imaging (MRI) has been shown to correlate with lumbar skeletal muscle mass [6], the latter serving as a surrogate marker of sarcopenia in several solid cancers [6]. In previous investigations, low TMT was associated with worse survival in patients with newly diagnosed as well as recurrent glioblastoma [7, 8, 9, 10, 11, 12, 13, 14, 15, 16, 17]. However, previous investigations suffered from methodological shortcomings such as insufficient sample size, heterogeneous patient cohorts, missing key molecular and clinical data (*MGMT* promoter methylation status, isocitrate dehydrogenase—*IDH*—status, information on adjuvant systemic treatment), missing longitudinal assessments of TMT, and missing established and uniform TMT cutoff values.

In accordance with a recommendation of the European Working Group on Sarcopenia in Older People (EWGSOP) [18] Furtner et al. proposed sex‐specific TMT cutoff values and demonstrated—on the basis of the CENTRIC EORTC 26071‐22072 study [19] and the CORE study [20]—that risk of sarcopenia at baseline as well as TMT loss over time was associated with unfavorable survival [14]. These sex‐specific cutoff values (≤ 6.3 mm in male patients; ≤ 5.2 mm in female patients) were validated in a real‐life analysis of newly diagnosed glioblastoma patients by Broen et al. [15]. However, this analysis did not provide a uniformly treated glioblastoma cohort and lacked also longitudinal TMT assessments.

In our multicentric analysis, featuring data from five neuro‐oncology centers in Germany, we investigated the prognostic value of TMT using a homogeneous cohort of newly diagnosed *MGMT* promoter methylated glioblastoma patients. These patients were uniformly treated under real‐life conditions, having received maximum‐safe surgical resection, radiotherapy, and the combination of CCNU and temozolomide according to the CeTeG/NOA‐09 trial [3]. A balanced multicentric control cohort of newly diagnosed *MGMT* promoter methylated glioblastoma patients, uniformly treated under real‐life conditions with maximum‐safe surgical resection, radiotherapy, and temozolomide according to the EORTC‐NCIC‐26981‐22981/CE.3 trial served as a comparison [21].

## 2 Materials and Methods

### 2.1 Study Design

For this multicentric analysis, we gathered clinicopathological as well as radiographical data from newly diagnosed glioblastoma patients between May 2012 and August 2020 at five neuro‐oncology centers in Germany (University Hospital Essen, University Hospital Leipzig, University Hospital Münster, University Hospital Regensburg, University Hospital Würzburg). The following selection criteria had to be met:

1. Adult patients with newly diagnosed glioblastoma.
2. Histopathologically confirmed methylation of the *MGMT* promoter, which was determined locally through quantitative methylation‐specific polymerase chain reaction (PCR) or deoxyribonucleic acid (DNA) pyrosequencing.
3. *IDH* wild‐type status.
4. First‐line treatment with maximum‐safe surgical resection, radiotherapy, and the combination of temozolomide and CCNU for at least six weeks (one course). Concomitant and adjuvant treatments with CCNU plus temozolomide were allowed as well as initially concomitant single‐drug treatment with temozolomide followed by adjuvant treatment with CCNU plus temozolomide and combined treatment with tumor treating fields (TTFields).

All data for the above mentioned cohort (CeTeG cohort) were obtained in an anonymized format within the framework of routine clinical assessments. The radiographic data was shared in the form of Digital Imaging and Communications in Medicine (DICOM) files. A radiologist (board‐certified, 13 years of experience, CM) not involved in the collection of data determined the initial extent of tumor resection and reviewed all MRI scans for tumor recurrence, putative pseudoprogression, and treatment response in accordance with the updated response assessment criteria for high‐grade gliomas [22, 23]. Archived MRI scans were available at 8–12 weeks intervals.

To demonstrate a broad, treatment‐independent practicability of TMT as a prognostic parameter in *MGMT* promoter‐methylated newly diagnosed glioblastoma, we identified a multicentric control cohort of *n* = 60 patients, treated with temozolomide and/or TTFields. This control cohort (Stupp cohort) was balanced to the CeTeG cohort regarding canonical clinical features such as histopathological diagnosis, age, KPS, sex, *MGMT* promoter status, and additional treatments (such as TTFields) in first‐line treatment. The Stupp cohort consisted of glioblastoma patients newly diagnosed between February 2014 and December 2020 at five neuro‐oncology centers in Germany (University Hospital Essen, University Hospital Leipzig, University Hospital Münster, University Hospital Regensburg, University Hospital Würzburg). The same selection criteria were applied to the Stupp cohort and to the CeTeG cohort; the only exception was that combination treatment with CCNU was not permitted and treatment with temozolomide had to be given for at least four weeks (one course).

This analysis was approved by the local ethics committee at the University of Duisburg‐Essen (reference number: 20‐9431‐BO; date of approval: August 12, 2020).

### 2.2 Baseline Assessment of TMT

For every patient from the two cohorts, preoperative (to the initial brain tumor surgery) as well as postoperative (to the initial brain tumor surgery) MR images were retrospectively retrieved from the participating neuro‐oncology centers. The baseline assessment of TMT was performed using the postoperative (conducted within 72 h after the initial brain surgery) MR image; in patients who could not be evaluated for postoperative TMT—for example, because of partial depiction of both temporal muscles, prior therapeutic intervention such as incision involving the temporal muscles, poor imaging quality—the preoperative MR image was used for baseline TMT assessment.

**FIGURE 1:**
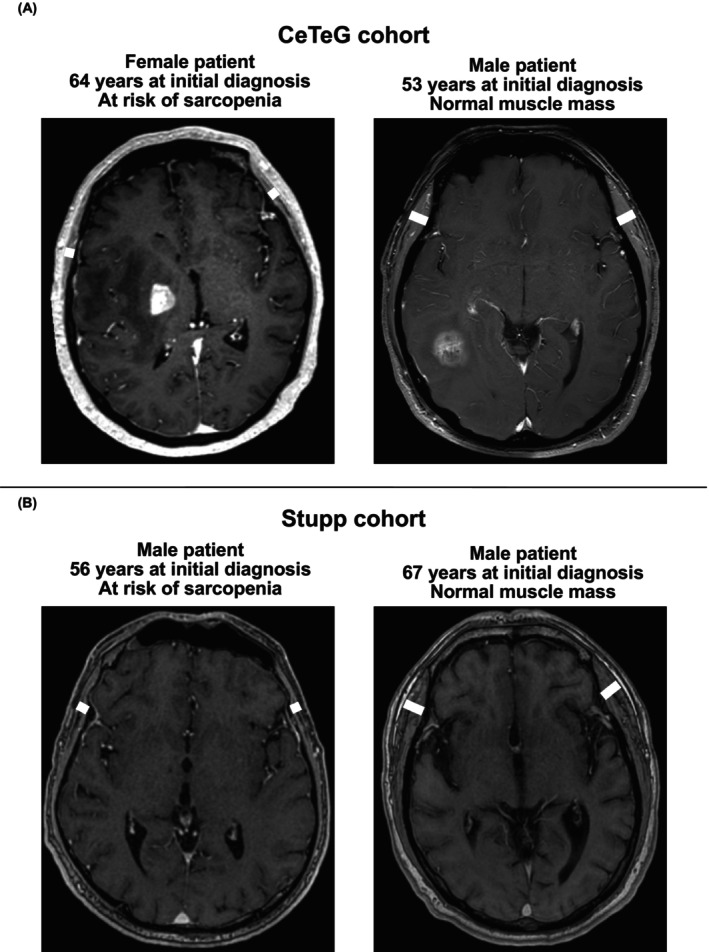
Examples of TMT assessment for patients from the CeTeG and Stupp cohorts. (A and B) indicate examples of patients at risk of sarcopenia (left) and patients with normal muscle mass (right) from the CeTeG (above) and Stupp cohort (below). TMT measurement was assessed on axial T1‐weighted contrast‐enhanced cranial MRI. The white lines indicate the exact calculation of TMT for both sides. MRI: Magnetic resonance imaging; TMT: Temporal muscle thickness.

MR examinations of the skull were performed at five neuro‐oncology centers in Germany (University Hospital Essen, University Hospital Leipzig, University Hospital Münster, University Hospital Regensburg, University Hospital Würzburg) on ≥ 1.5 Tesla MR scanners with transmit/receive head coils. MR sequences at each center included T1 TSE, T2 FLAIR, DWI, and 3D T1 MPRAGE MR sequences after intravenous application of a gadolinium‐containing contrast agent. Temporal muscle thickness was determined on transverse T1 weighted images (Figure 1) by a board‐certified neuroradiologist without access to clinical patient data (CM). The plane was set parallel to the anterior commissure–posterior commissure line; measurements had to be performed on both sides at the level of the orbital roof (cranio‐caudal landmark) and the lateral sulcus (anterior–posterior landmark) perpendicular to the long axis of the temporal muscle. After TMT determination for both sides, the mean TMT was calculated and used for further analysis. In case TMT measurement was not possible on one side (due to prior surgical intervention), only the TMT value of the opposite side was taken into account. Figure 1 delivers an example of TMT measurement according to the above‐described procedure. For the identification of patients at risk of sarcopenia, the sex‐specific TMT cutoff values proposed by Furtner et al. [14] (≤ 6.3 mm in male patients; ≤ 5.2 mm in female patients) were implemented.

### 2.3 Longitudinal Assessment of TMT

For longitudinal assessment of TMT, the first follow‐up MRI after completion of radiotherapy (3–6 weeks after radiotherapy) was used. In case of by any means (partial depiction of both temporal muscles, prior therapeutic intervention such as incision involving the temporal muscles, poor imaging quality) disabling TMT evaluation, the patients were excluded from longitudinal analysis. The ratio of the follow‐up TMT and the baseline TMT was calculated and denoted as relTMT. In accordance with the analysis of Furtner et al. [14], patients were subdivided into three subgroups: No TMT loss (relTMT ≥ 100%), mild TMT loss (relTMT = 99%–90%), and severe TMT loss (relTMT < 90%). For further longitudinal analysis, patients were subdivided according to the quartiles of the relTMT values; we also used a receiver operating characteristic (ROC) analysis to identify the optimal cutoff for relTMT stratification concerning its impact on overall survival.

### 2.4 Statistical Analysis

We presented clinicopathological patient characteristics descriptively in a tabular format. For the comparison of feature distribution within the analyzed subgroups, we used the Mann–Whitney U test (continuous variables) and the Fisher's exact test (categorical variables). We selected cutoff levels for age groups, KPS, and extent of resection according to the analysis previously performed by Furtner et al. [14] in order to facilitate comparison. To estimate the survival function from lifetime data, we used the Kaplan–Meier estimator. In case progression or death had not occurred at the time of data analysis, the corresponding patient data was censored for further survival analysis. Multivariable Cox regression models were used to determine independently significant predictors for PFS and OS. For data visualization, GraphPad Prism version 10.0.3 (GraphPad Software Inc., San Diego, USA) and Affinity Designer version 1.10.6 (Serif Europe, West Bridgford, UK) were used.

## 3 Results

### 3.1 Patient Characteristics

In total, *n* = 66 patients in the CeTeG cohort and *n* = 60 patients in the Stupp cohort met the inclusion criteria and were included in this analysis. The basic clinical characteristics—such as age at diagnosis, KPS, sex, and initial extent of resection—were similarly distributed between the CeTeG and Stupp cohorts. In each cohort, *n* = 10 patients at risk of sarcopenia (CeTeG cohort: 15%; Stupp cohort: 17%) at baseline could be identified. Baseline TMT values ranged from 4.3 mm to 11.9 mm (median: 8.9 mm) in the CeTeG cohort and from 5.2 mm to 13.3 mm (median: 7.8 mm) in the Stupp cohort. In a total of *n* = 4 patients (two from the Stupp cohort and two from the CeTeG cohort), longitudinal TMT was not assessable; in *n* = 6 patients (all from the Stupp cohort), the preoperative MR image was used for baseline TMT assessment. In the course of disease, the fraction of patients with stable or increasing TMT (relTMT ≥ 100%) was lower in the CeTeG cohort as opposed to the Stupp cohort (CeTeG cohort: *n* = 17, 26%; Stupp cohort: *n* = 24, 40%; *p* = 0.1273). By contrast, the fraction of patients with a relevant TMT decline (relTMT < 90%) was higher in the CeTeG cohort as opposed to the Stupp cohort (CeTeG cohort: *n* = 10, 15%; Stupp cohort: *n* = 6, 10%; *p* = 0.4326). Those patients without TMT decline in the course of disease (relTMT ≥ 100%) predominantly showed a KPS ≥ 90% (*n* = 12/17 [71%] in the CeTeG cohort; *n* = 14/24 [58%] in the Stupp cohort; *p* = 0.5194). From all patients with normal muscle mass at baseline, *n* = 65/102 (64%) had TMT decline (relTMT < 90% plus relTMT 90%–99%) in the course of disease (CeTeG cohort: *n* = 38/54 [70%]; Stupp cohort: *n* = 27/48 [56%]); from all patients at risk of sarcopenia at baseline *n* = 16/20 (80%) had TMT decline (relTMT < 90% plus relTMT 90%–99%) in the course of disease (CeTeG cohort: *n* = 9/10 [90%]; Stupp cohort: *n* = 7/10 [70%]). Absolute longitudinal TMT decrease ranged from 0.1 mm to 2.2 mm (median: 0.3 mm); absolute longitudinal TMT increase ranged from 0.1 mm to 1.3 mm (median: 0.3 mm). A comparative synopsis of detailed patient characteristics from both multicentric cohorts is given by Table 1. In Tables 2 and 3 a comparison of clinical feature distribution among patients with normal muscle mass and patients at risk of sarcopenia is given for both cohorts.

**TABLE 1:** Patient characteristics of the CeTeG and Stupp cohorts.

	CeTeG cohort (*n* = 66)	Stupp cohort (*n* = 60)	*p*
Age at diagnosis, *n*			
≥ 50 years	49 (74%)	51 (85%)	0.1861
< 50 years	17 (26%)	9 (15%)	
Karnofsky performance score, *n*			
≥ 90%	43 (65%)	33 (55%)	0.2770
< 90%	23 (35%)	27 (45%)	
Sex, *n*			
Men	36 (64%)	34 (57%)	0.8587
Women	30 (46%)	26 (43%)	
Extent of resection, *n*			
Partial resection or biopsy	40 (61%)	40 (67%)	0.5789
Complete resection	26 (39%)	20 (33%)	
TMT at baseline, *n*			
At risk of sarcopenia	10 (15%)	10 (17%)	
Normal muscle mass	56 (85%)	50 (83%)	
Longitudinal TMT, *n*			
relTMT ≥ 100%	17 (26%)	24 (40%)	
relTMT 90%–99%	37 (56%)	28 (47%)	
relTMT < 90%	10 (15%)	6 (10%)	
NA	2 (3%)	2 (3%)	
Follow‐up in months, median (range)	34 (5–102)	23 (5–108)	

Abbreviations: NA: Not assessable; TMT: Temporal muscle thickness.

**TABLE 2:** Clinical feature distribution among patients with normal muscle mass and patients at risk of sarcopenia in the CeTeG cohort.

	Normal muscle mass (*n* = 56)	At risk of sarcopenia (*n* = 10)	*p*
Age at diagnosis, *n*			
≥ 50 years	41 (73%)	8 (80%)	0.9999
< 50 years	15 (27%)	2 (20%)	
Karnofsky performance score, *n*			
≥ 90%	39 (70%)	4 (40%)	0.0847
< 90%	17 (30%)	6 (60%)	
Sex, *n*			
Men	32 (57%)	4 (40%)	0.4924
Women	24 (43%)	6 (60%)	
Extent of resection, *n*			
Partial resection or biopsy	34 (61%)	6 (60%)	0.9999
Complete resection	22 (39%)	4 (40%)	
Longitudinal TMT, *n*			
relTMT ≥ 100%	16 (29%)	1 (10%)	
relTMT 90%–99%	30 (53%)	7 (70%)	
relTMT < 90%	8 (14%)	2 (20%)	
NA	2 (4%)	0 (−)	
Follow‐up in months, median (range)	35 (11–102)	17 (5–47)	

Abbreviations: NA: Not assessable; TMT: Temporal muscle thickness.

**TABLE 3:** Clinical feature distribution among patients with normal muscle mass and patients at risk of sarcopenia in the Stupp cohort.

	Normal muscle mass (*n* = 50)	At risk of sarcopenia (*n* = 10)	*p*
Age at diagnosis, *n*			
≥ 50 years	41 (82%)	10 (100%)	0.3328
< 50 years	9 (18%)	0 (−)	
Karnofsky performance score, *n*			
≥ 90%	28 (56%)	5 (50%)	0.7422
< 90%	22 (44%)	5 (50%)	
Sex, *n*			
Men	27 (54%)	7 (70%)	0.4905
Women	23 (46%)	3 (30%)	
Extent of resection, *n*			
Partial resection or biopsy	35 (70%)	5 (50%)	0.2776
Complete resection	15 (30%)	5 (50%)	
Longitudinal TMT, *n*			
relTMT ≥ 100%	21 (42%)	3 (30%)	
relTMT 90%–99%	23 (46%)	5 (50%)	
relTMT < 90%	4 (8%)	2 (20%)	
NA	2 (4%)	0 (−)	
Follow‐up in months, median (range)	29 (6–108)	18 (5–25)	

Abbreviations: NA: Not assessable; TMT: Temporal muscle thickness.

When excluding patients for whom longitudinal MRI examinations were lacking from the CeTeG and Stupp cohorts, similar survival times (median progression‐free survival ‐ mPFS ‐ and median overall survival ‐ mOS) were observed (CeTeG cohort versus Stupp cohort—mPFS: 14.8 months versus 10.2 months, *p* = 0.6612; mOS: 33.7 months versus 21.3 months, *p* = 0.0418; normal muscle mass versus sarcopenia in the CeTeG cohort—mPFS: 16.5 months versus 6.8 months, *p* = 0.0012; mOS: 67.4 months versus 16.7 months, *p* = 0.0057; normal muscle mass versus sarcopenia in the Stupp cohort—mPFS: 11.3 months versus 7.7 months, *p* = 0.0276; mOS: 27.2 months versus 17.4 months, *p* = 0.0010).

### 3.2 Association of TMT at Baseline With Survival

**FIGURE 2:**
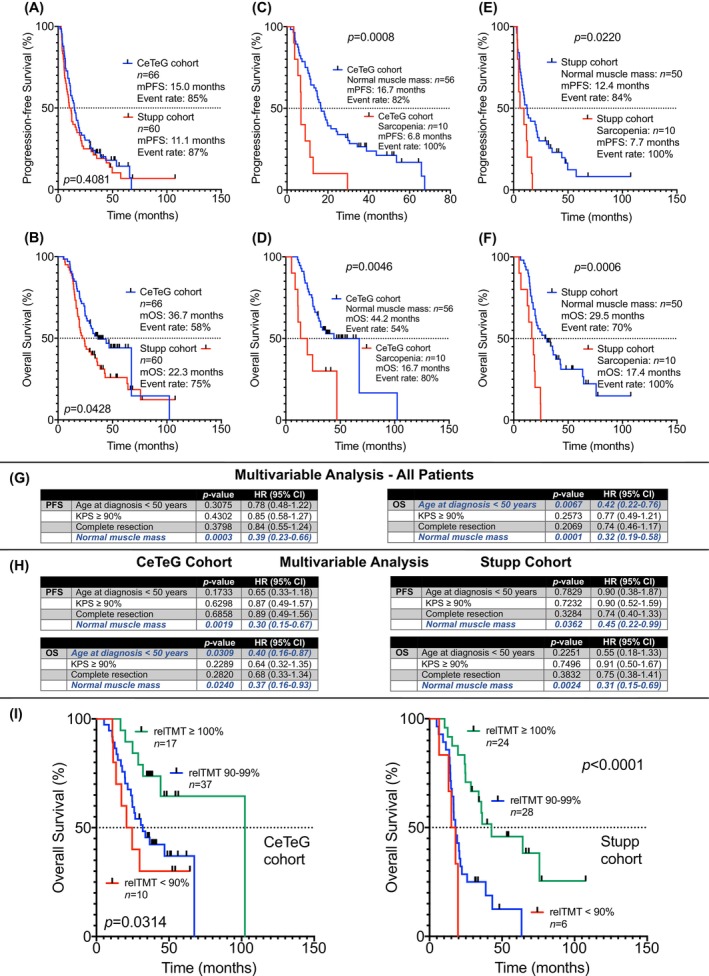
Association of TMT with survival. (A and B) comparatively shows mPFS and mOS of the CeTeG and Stupp cohorts. In both cohorts, patients with sex‐specific TMT‐based normal muscle mass at baseline had significantly prolonged survival (C–F). (G) shows the results of a multivariable Cox regression analysis including all patients and featuring the canonical clinical factors with known prognostic relevance. (H) depicts the results of a multivariable Cox regression analysis including the CeTeG patients (left) and the Stupp patients (right). For both cohorts, mOS was longest in patients who did not experience a decline of TMT (relTMT ≥ 100%) in the course of the disease and shortest in patients who experienced a relevant decline in TMT (relTMT < 90%) in the disease course (I). mOS: Median overall survival; mPFS: Median progression‐free survival; TMT: Temporal muscle thickness.

Median progression‐free survival (mPFS) in the CeTeG cohort was 15.0 months, and median overall survival (mOS) was 36.7 months. In the Stupp cohort, mPFS was 11.1 months, and mOS was 22.3 months (Figure 2A,B). In each of the cohorts, patients with normal TMT performed significantly better in terms of PFS and OS than patients with reduced TMT (mPFS for the CeTeG cohort: 16.7 months versus 6.8 months, HR: 2.973, 95% CI: 1.051–8.405, *p* = 0.0008; mOS for the CeTeG cohort: 44.2 months versus 16.7 months, HR: 2.912, 95% CI: 0.9250–9.170, *p* = 0.0046; mPFS for the Stupp cohort: 12.4 months versus 7.7 months, HR: 2.152, 95% CI: 0.8683–5.333, *p* = 0.0220; mOS for the Stupp cohort: 29.5 months versus 17.4 months, HR: 2.912, 95% CI: 1.092–9.044, *p* = 0.0006; Figure 2C–F).

### 3.3 Multivariable Analysis

In a multivariable Cox regression analysis including all patients—both from the CeTeG cohort as well as from the Stupp cohort—and featuring the canonical clinical factors with known prognostic relevance (age at diagnosis, KPS, initial extent of resection), normal muscle mass at baseline (or lack of sarcopenia at baseline) emerged as the only statistically significant and independent prognostic marker regarding PFS (HR: 0.39, 95% CI: 0.23–0.66; *p* = 0.0003). Concerning OS, besides normal muscle mass at baseline (HR: 0.32, 95% CI: 0.19–0.58; *p* = 0.0001) an initial age at diagnosis of < 50 years also emerged as a statistically significant prognostic marker (HR: 0.42, 95% CI: 0.22–0.76; *p* = 0.0067) (Figure 2G).

In a multivariable Cox regression analysis including the patients from the CeTeG cohort, normal muscle mass at baseline (or lack of sarcopenia at baseline) emerged as the only statistically significant and independent prognostic marker regarding PFS (HR: 0.30, 95% CI: 0.15–0.67; *p* = 0.0019). Concerning OS, besides normal muscle mass at baseline (HR: 0.37, 95% CI: 0.16–0.93; *p* = 0.0240) also an initial age at diagnosis of < 50 years emerged as a statistically significant prognostic marker (HR: 0.40, 95% CI: 0.16–0.87; *p* = 0.0309) (Figure 2H).

In a multivariable Cox regression analysis including the patients from the Stupp cohort, normal muscle mass at baseline (or lack of sarcopenia at baseline) emerged as the only statistically significant and independent prognostic marker regarding PFS (HR: 0.45, 95% CI: 0.22–0.99; *p* = 0.0362) and OS (HR: 0.31, 95% CI: 0.15–0.69; *p* = 0.0024) (Figure 2H).

### 3.4 Association of Longitudinal TMT With Survival

Longitudinal analysis was possible in *n* = 64 patients of the CeTeG cohort and *n* = 58 patients of the Stupp cohort. For both cohorts (the CeTeG cohort as well as the Stupp cohort), mOS was longest in patients who did not experience a decline in TMT (relTMT ≥ 100%; mOS for CeTeG cohort: 102.3 months; mOS for Stupp cohort: 42.7 months) in the course of the disease. In contrast, for both cohorts mOS was shortest in patients who experienced a relevant decline in TMT (relTMT < 90%; mOS for the CeTeG cohort: 22.7 months; mOS for the Stupp cohort: 16.5 months) in the disease course. The observed differences in survival times dependent on relTMT were statistically significant (CeTeG cohort: *p* = 0.0314; Stupp cohort: *p* < 0.0001; Figure 2I).

**FIGURE 3:**
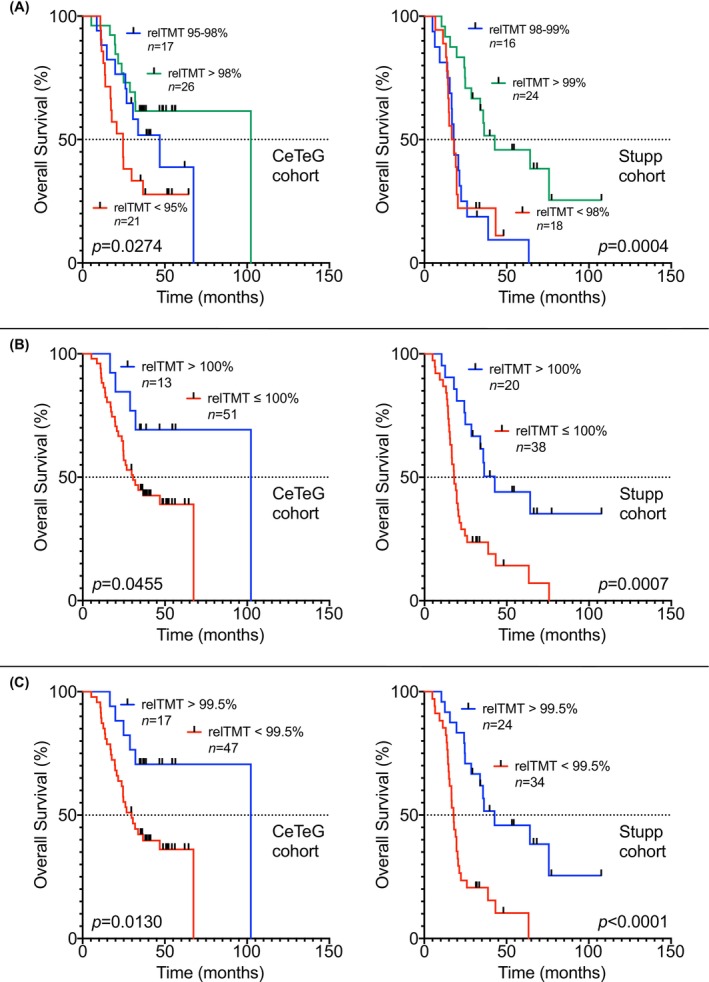
Longitudinal analysis of TMT with survival. (A) shows the survival of patients from both cohorts subgrouped by first and third quartiles of relTMT values. (B) depicts the survival of patients from both cohorts stratified by increase versus decline of relTMT in the disease course. (C) illustrates the survival of patients from both cohorts subdivided by receiver operating characteristic (ROC) defined relTMT cutoff (Youden's index). In all subgroup analyses, higher relTMT values in the disease course were associated with statistically significant prolonged survival. TMT: Temporal muscle thickness.

For further analysis, we calculated the survival of patient subgroups stratified by the first and third quartile of relTMT values (Figure 3A), stratified by increase versus decline of relTMT in the disease course (Figure 3B) and by ROC‐defined relTMT cutoff (Youden's index) for both cohorts (Figure 3C). In all subgroup analyses higher relTMT values in the disease course were associated with statistically significant prolonged survival. In both cohorts, mOS was highest in patients with relTMT values above the third quartile (mOS: 102.3 months for the CeTeG cohort and 42.7 months for the Stupp cohort; Figure 3A), in patients with gain of relTMT in the disease course (mOS: 102.3 months for the CeTeG cohort and 42.7 months for the Stupp cohort; Figure 3B), and in patients with relTMT values above the Youden's index (mOS: 102.3 months for the CeTeG cohort and 42.7 months for the Stupp cohort; Figure 3C).

## 4 Discussion

In this multicenter real‐world analysis, we demonstrated that temporal muscle thickness at baseline and over time serves as a robust prognostic biomarker in newly diagnosed *MGMT* promoter methylated glioblastoma patients treated with radiochemotherapy. Across both the CeTeG cohort and the Stupp (±TTFields) cohort, reduced baseline temporal muscle thickness indicative of sarcopenia was significantly associated with poorer progression‐free and overall survival. This finding confirms prior reports that linked low temporal muscle thickness with unfavorable outcomes in glioblastoma [7, 8, 9, 10, 11, 12, 13, 14, 15, 16, 17]. Moreover, our study advances this field by using established sex‐specific cutoffs to define sarcopenia, analyzing treatment‐homogeneous cohorts, and incorporating key molecular information (i.e., *MGMT* status).

In this analysis, we used two balanced multicentric newly diagnosed *MGMT* promoter methylated glioblastoma cohorts uniformly treated under real‐life conditions with maximum‐safe surgical resection, radiotherapy, and either the combination of CCNU and temozolomide according to the CeTeG/NOA‐09 trial [3] or single‐drug temozolomide according to the EORTC‐NCIC‐26981‐22981/CE.3 trial [21]. The combination of CCNU and temozolomide is currently a frequently used treatment for *MGMT* promoter methylated glioblastoma patients. So far, data on TMT in patients exclusively treated with CCNU and temozolomide as well as exclusively newly diagnosed with *MGMT* promoter methylated glioblastoma are not available. To evaluate whether an observed TMT effect is due to the combination of CCNU and temozolomide treatment or due to *MGMT* promoter methylation per se, which is the most important prognostically relevant marker in glioblastoma, it was important to exclusively include *MGMT* promoter methylated patients in both cohorts—CeTeG as well as Stupp. Upon implementation of the recently proposed sex‐specific cutoff values by Furtner et al. [14], we observed rates of patients at risk of sarcopenia (TMT ≤ 6.3 mm in male patients, and ≤ 5.2 mm in female patients) similar to those reported by Broen et al. in a TMT analysis investigating newly diagnosed glioblastoma patients (CeTeG cohort: 15%; Stupp cohort: 17%; analysis by Broen et al.: 16%) [15]. Unlike some previous investigations, this analysis provides a clinicopathologically uniform cohort of newly diagnosed *MGMT* promoter methylated glioblastoma patients with exclusively *IDH* wild‐type tumors and a balanced control cohort. The use of a molecularly and clinically well‐defined glioblastoma cohort in our analysis aiming to minimize potential selection bias is an important strength of this work compared to previous works evaluating TMT in the field, which suffered from methodological shortcomings, such as heterogeneous patient cohorts and missing key molecular and clinical data (*MGMT* promoter methylation status, *IDH* status, information on adjuvant systemic treatment). In the field of neuro‐oncology, it is particularly important to study clinically as well as molecularly homogeneous populations to obtain the most meaningful results with the lowest possible bias. On this basis, we validated under real‐life conditions the sex‐specific TMT cutoff values proposed by Furtner et al. [14]. Unlike all other previous investigations, both preoperative and postoperative MR images (in case of disabled TMT evaluation in the postoperative MR images) were available for precise TMT calculations. Analogous to Furtner et al. [14] and unlike Broen et al. [15], we assessed not only baseline TMT but also longitudinal TMT alteration. Our analysis indicates sarcopenia at baseline is associated with adverse survival, and TMT stabilization or decrease over the disease course is associated with favorable or unfavorable survival, respectively. It is reasonable to assume that sarcopenia reflects the degree of catabolic activity and tumor cachexia in general, whose main aspect is continuous muscle wasting. Cachectic patients experience various symptoms that may affect several organ functions and decrease quality of life and worsen patients' prognosis [24]. Of note, based on a literature review in MEDLINE using the search terms “Glioblastoma” and “Sarcopenia” we identified a previous work from Morshed et al., in which several markers of sarcopenia (including the masseter and temporal muscle diameters) were assessed in a subgroup of elderly glioblastoma patients; in this work, masseter diameter on preoperative imaging was associated with shorter overall survival [25]. In this context, a comparative survival analysis with a matched healthy control cohort is lacking and would be of interest to clarify the question of to what extent elderly age per se is associated with sarcopenia.

Of note, there was a non‐significant correlation between KPS and baseline TMT in the CeTeG cohort that was not observed in the Stupp cohort. CeTeG patients with a KPS ≥ 90% tended to have a higher TMT than those with KPS < 90%. Additionally, those patients without TMT decline in the course of disease (reTMT ≥ 100%) predominantly showed a KPS ≥ 90%, specifically in the CeTeG cohort. This hints at a link between overall fitness and muscle preservation, though in multivariable analysis this did not impact the prognostic effect of baseline TMT. The relationship between performance status and temporal muscle mass merits further study. Importantly, TMT demonstrated prognostic value independent of CCNU/temozolomide treatment.

Under real‐life conditions, the overall survival of the CeTeG cohort did not reach that of the original CeTeG/NOA‐09 trial (36.7 versus 48.1 months) [3]. However, overall survival was significantly longer in our CeTeG cohort than in the Stupp cohort (36.7 versus 22.3 months). Based on sex‐specific TMT cutoffs, selecting CeTeG patients with normal baseline muscle mass extended overall survival to 44.2 months, nearing the original CeTeG/NOA‐09 trial estimates [3].

This study has some limitations. The small sample sizes and generally low muscle mass values may contribute to measurement inaccuracy. Aiming to optimize the assessment of TMT, future studies should be performed on uniform MRI devices using uniform MRI protocols. TMT evaluators should also receive standardized training in the assessment of TMT with particular focus on potential sources of error; furthermore, the development and use of automated AI‐based analysis software for the calculation of TMT would be possible and help standardize TMT calculation and comparability. It would also be helpful to have TMT values calculated by two different board‐certified neuro‐radiologists blinded to clinical patient characteristics and outcome measures accounting for bias associated with interrater variability. The retrospective design and lack of TMT measurement validation also limit conclusions. Nevertheless, we validated the prognostic value of sex‐specific TMT cutoffs and longitudinal TMT alteration proposed by Furtner et al. [14]. In principle, TMT may help stratify glioblastoma patients into those with a relatively poor and those with a relatively favorable prognosis. In addition to age and KPS, TMT can be important in the selection of therapeutic options, especially for intensified protocols (e.g., the combination of CCNU and temozolomide). Since reduced temporal muscle thickness is usually observed in patients with decreased nutritional intake, it could be reasonable to consider TMT as a biomarker for nutritional status and thus a surrogate for clinical decline that may not be detected by the physician's impression on the patients' performance status or KPS. Whether reversal of TMT decline by improving patients' nutrition over time results in improved survival needs to be tested. In summary, the assessment of TMT may be useful in the attempt to personalize oncological treatment options in daily neuro‐oncological patient care. Of course, evaluation of the above mentioned potential TMT applications in daily oncological practice requires investigation in separate follow‐up studies.

## Author Contributions

**Lazaros Lazaridis:** conceptualization (lead), data curation (lead), formal analysis (lead), project administration (lead), visualization (lead), writing – original draft (lead), writing – review and editing (lead). **Christoph Moenninghoff:** data curation (lead), writing – review and editing (supporting). **Elisabeth Bumes:** data curation (supporting), writing – review and editing (supporting). **Dorothee Cäcilia Spille:** data curation (supporting), writing – review and editing (supporting). **Michael Müther:** data curation (supporting), writing – review and editing (supporting). **Tim Schulz:** data curation (supporting), writing – review and editing (supporting). **Sina Heider:** data curation (supporting), writing – review and editing (supporting). **Sarina Agkatsev:** writing – review and editing (supporting). **Teresa Schmidt:** writing – review and editing (supporting). **Tobias Blau:** writing – review and editing (supporting). **Christoph Oster:** writing – review and editing (supporting). **Walter Stummer:** writing – review and editing (supporting). **Almuth Friederike Kessler:** writing – review and editing (supporting). **Clemens Seidel:** writing – review and editing (supporting). **Oliver Grauer:** writing – review and editing (supporting). **Peter Hau:** writing – review and editing (supporting). **Yahya Ahmadipour:** writing – review and editing (supporting). **Ulrich Sure:** writing – review and editing (supporting). **Kathy Keyvani:** writing – review and editing (supporting). **Ulrich Herrlinger:** writing – review and editing (supporting). **Christoph Kleinschnitz:** writing – review and editing (supporting). **Martin Stuschke:** writing – review and editing (supporting). **Nika Guberina:** writing – review and editing (supporting). **Ken Herrmann:** writing – review and editing (supporting). **Cornelius Deuschl:** writing – review and editing (supporting). **Björn Scheffler:** writing – review and editing (supporting). **Sied Kebir:** conceptualization (lead), data curation (supporting), formal analysis (lead), project administration (lead), supervision (lead), visualization (lead), writing – review and editing (lead). **Martin Glas:** conceptualization (lead), project administration (lead), supervision (lead), writing – review and editing (lead).

## Ethics Statement

This analysis was approved by the local institutional review board at the University of Duisburg‐Essen (reference number: 20‐9431‐BO; date of approval: August 12, 2020). Written informed consent was waived by the institutional review board due to the retrospective nature of the analysis. All data were anonymized before inclusion in the analysis. No sex‐based or racial/ethnic‐based differences were present in this analysis.

## Conflicts of Interest

Lazaros Lazaridis received honoraria and travel support from Novocure. Michael Müther received honoraria and travel support from Medac. Teresa Schmidt received honoraria and travel support from Novocure. Christoph Oster received honoraria and travel support from Novocure. Almuth Friederike Kessler received travel support and research grants from Novocure. Clemens Seidel received honoraria for lectures, consultation, or advisory board participation from the following for‐profit companies: AbbVie, Bristol‐Myers Squibb, HRA Pharma, Medac, Novocure, Roche, and Seagen. Peter Hau received honoraria from Bayer, Medac, Novocure, and Seagen; travel support from Novocure and Medac. Ulrich Herrlinger received lecture and/or advisory board honoraria from Medac, Noxxon, AbbVie, Bayer, Janssen, and Karyopharm. Björn Scheffler is supported by the German Cancer Consortium (DKTK). Sied Kebir received honoraria and travel support from Novocure. Martin Glas reports honoraria from Roche, Novartis, UCB, AbbVie, Daiichi Sankyo, Novocure, Bayer, Janssen‐Cilag, Medac, Merck, Kyowa Kirin, travel support from Novocure and Medac, and a research grant from Novocure. All remaining authors have declared no conflicts of interest.

## Data Availability

The data generated in this analysis are available within the article. Further information is available upon reasonable request from the corresponding author.
